# Diagnostic flow for all patients referred with non-specific symptoms of cancer to a diagnostic centre in Denmark: A descriptive study

**DOI:** 10.1080/13814788.2023.2296108

**Published:** 2024-01-05

**Authors:** Christina Sadolin Damhus, John Brandt Brodersen, Gunnar Lauge Nielsen

**Affiliations:** a The Centre of General Practice, Department of Public Health, University of Copenhagen, Copenhagen, Denmark; b The Research Unit for General Practice in Region Zealand, Denmark; c Department of Community Medicine, General Practice Research Unit, Faculty of Health Sciences, UiT, The Arctic University of Norway, Tromsø, Norway; d Department of Internal Medicine, Aalborg University Hospital, Aalborg, Denmark; e Department of Clinical Medicine, Aalborg University Hospital, Aalborg, Denmark

**Keywords:** Non-specific symptoms, diagnostic centre, cancer patient pathway, diagnoses

## Abstract

**Background:**

Since 2012, Cancer Patient Pathways for Non-specific Symptoms and Signs of Cancer (NSSC-CPP) have been implemented in Scandinavia and UK.

**Objectives:**

This study aimed to describe the diagnostic flow for all patients referred from 1 January to 30 June 2020 to the NSSC-CPP in the Diagnostic Centre in Farsø (DC-F), Denmark.

**Methods:**

During the study period, we prospectively recorded information on the diagnostic flow, including: pathway trajectory, symptoms and findings leading to referral, diagnostic procedures and diagnoses at the end of DC Farsø work-up and within 6-months for all patients referred to the NSSC-CPP in DC Farsø using electronic patient files and the Danish National Patient Registry (DNPR).

**Results:**

Of the 314 referrals to DC Farsø, 227 had diagnostic work-up in DC Farsø, the remaining were redirected to other CPPs (*n* = 11), outpatient clinics (*n* = 45) or redirected to general practice (*n* = 25). Of total referrals, 25 (8%) received a malignant diagnosis, 20 (6%) a non-malignant but clinically relevant diagnosis with initiation of treatment, 16 (5%) a non-malignant diagnosis but no treatment needed and in 253 (81%) referrals no severe new condition was diagnosed. Two (1%) additional malignancies were diagnosed within a 6-month follow-up period.

**Conclusion:**

By tracking all patients referred to the NSSC-CPP in DC Farsø, including those redirected, this is the first study to describe the diagnostic flow for all patients referred to a diagnostic centre in Denmark. This knowledge is important for further organisation and planning of the NSSC-CPP.

## KEY MESSAGES

Eight percent of NSSC-CPP referrals yielded cancer diagnoses, with two additional cancers diagnosed in a 6-month follow-up.

Over one-fourth of referrals to NSSC-CPP were redirected to other departments or general practitioners.

This study outlines the diagnostic flow for all NSSC-CPP-referred patients, offering unique insights crucial for comparing European diagnostic practices.

## Introduction

Early diagnosis of cancer is a priority of governments in the Global North, which in the United Kingdom (UK) led to the implementation of two-week wait referrals (2WW) to support general practitioners (GPs) in fast detection of cancer [1]. Similarly, Cancer Patient Pathways (CPPs) or clinical guidelines to expedite the investigation, diagnosis, and treatment of symptomatic individuals have been introduced in Norway [2], Sweden [3], United Kingdom [4], Spain and New Zealand [5,6]. In Denmark, since 2007, patients with alarm symptoms of cancer can be referred to an Organ Specific Cancer Patient Pathway (OS-CCP), which is a standardised fast-track pathway established for 31 suspected cancer types [7]. However, more than half of patients with cancer present with vague or non-specific symptoms, such as unexplained weight loss, fatigue or anaemia, which do not qualify for an OS-CPP [8]. Consequently, in 2012, an additional CPP for non-specific symptoms and signs of cancer (NSSC-CPP), also referred to as the ‘diagnostic pathway,’ was implemented in Denmark and in 2015 copied to Norway and Sweden (Box 1) [2,3, 9]. Likewise, with inspiration from Denmark, UK, introduced in 2017 NSSC-CPPs, in different versions to reflect local healthcare systems and clinical priorities [10–15].

As far as we are aware, no studies have followed the trajectory of a consecutive cohort of patients first referred to the NSSC-CPP irrespective the setting in which the following work-up was carried out. This information is essential to document quality in the diagnostic centre’s decisions and assess the outcomes in patients referred to these centres. Therefore, this study aimed to describe the diagnostic flow, including pathway trajectory, symptoms and findings leading to referral, diagnostic procedures, diagnoses at the end of DC work-up and new malignant diagnoses within a six-month follow-up period for all patients referred from 1 January to 30 June 2020 to the NSSC-CPP in the Diagnostic Centre in Farsø (DC Farsø), Denmark.

Box 1. The Cancer Patient Pathway for Non-specific Symptoms and Signs of Cancer (NSSC-CPP) in Denmark: Setting, Guidelines and Practice

In the Danish healthcare system, general practitioners (GP) are gatekeepers to the secondary healthcare system and the CPPs and NSSC-CPPs are most often initiated by GPs [1]. The first mandatory step of the NSSC-CPP includes anamnesis, objective examination and a pre-specified blood panel. If deemed necessary diagnostic images as X-ray, ultrasound or CT scan may be added after this initial work-up. According to national guidelines, GPs can take this second step themselves or send patients directly to the hospital departments responsible for NSSC-CPP [2]. These departments, often referred to as diagnostic centres (DC), are responsible for all diagnostic steps, such as further diagnostic work-up or redirection of the patient [2]. Twenty-one diagnostic centres are implemented across the five health regions in Denmark where regional and intra-regional differences regarding organisational and clinical practice have been demonstrated in the diagnostic centres [4–6].

About 10% of patients referred to the NSSC-CPP in Denmark are diagnosed with cancer after completed workups and the remaining 90% receive either a diagnosis of another disease or no disease [1]. Previous studies in Denmark have described the characteristics of the NSSC-CPP referred population and focused on the risk of cancer, another serious disease, or mortality in this population [8–13]. However, these patients constitute a heterogeneous group and the work-up most often requires expertise from different medical specialities in the secondary health care sector [14]. Some patients referred to the NSSC-CPP are redirected by consultants in the diagnostic centre either to an OS-CPPs, investigation in other out-patient clinics or referred back to the general practitioner.

## Methods

### Study population

This study is a descriptive prospective study including all patients in the NSSC-CPP referred to the DC Farsø in North Denmark Region from 1 January to 30 June 2020. Data on symptoms and findings leading to referral and details on DC work-up were recorded daily on paper sheets by the DC Farsø doctors and later transferred to electronic files. Information about diagnoses obtained in the following six months was obtained from the Danish National Patient Registry (DNPR)[16]. For all patients rejected from DC investigation, data on outcomes were collected from a search in DNPR. Patients were identified and tracked in the registries by the unique personal identity number assigned to all Danish citizens at birth or immigration [17].

### DC Farsø setting

Since 2018, the DC Farsø has been responsible for work-up in all patients with non-specific symptoms in the catchment area of Aalborg University Hospital (Aalborg UH), with about 250,000 inhabitants. The staff in DC Farsø consists of three senior consultants, all with speciality in internal medicine and additional subspecialty in nephrology, gastroenterology, and hepatology. In 2018, 665 patients were referred to the NSSC-CPP in DC Farsø, which increased to 724 in 2021.

### Redirection and organisation of diagnostic workup in DC Farsø

Within the NSSC-CPP, the mandatory first step for the GP is to order predefined laboratory analyses. In cases where these blood tests do not give a clue to the direction of further work-up and where suspicion of malignancy is maintained, the GPs can either order a diagnostic X-ray, ultrasound or CT scan or refer the patient directly to the DC Farsø. If the GP decides to order a CT scan, the GP is urged to await a description before eventually referring to the DC Farsø. One of the three consultants in DC Farsø evaluates all referrals to the NSSC-CPP. Based on symptoms and findings as outlined in the referral letter, previous disease history with previous examinations, obtained by reviewing medical files in DNPR, the consultants make the decision on the following diagnostic process and if the patient needs to be directed to other units within the healthcare system, e.g. patients with microcytic anaemia are referred directly to OS-CPP for suspected colorectal cancer. This initial decision about selected trajectory is thus based on accessible data but without physical consultation.

### Diagnostic flow

The following outcomes constitute the diagnostic flow within this study and are described for the entire study population:

- **Pathway trajectory**
  - Redirection of referrals into OS-CPP, Acute hospital admission, Out-patient clinic, GP, DC Farsø
- **Symptoms and findings leading to referral**
  - List of predefined categories based on symptoms and findings that had been the most prevalent reasons for referral in the preceding years in DC Farsø: weight loss, fatigue, anaemia, abnormal laboratory values, abnormal x-ray, severe itching, sweats, no symptoms.
- **Diagnostic procedures**
  - CT scan, X-ray, ultrasound, MRI, gastroscopy, colonoscopy, sigmoidoscopy, capsule endoscopy.
- **New diagnoses at the end of DC Farsø work-up and within a 6-month follow-up period**
  - Malignant diagnosis, Non-malignant but clinically relevant diagnosis with initiation of treatment, Non-malignant diagnosis without initiation of treatment, No serious condition diagnosed (none of the above-referenced categories).

### Ethical considerations

In Denmark, approval from the Research Ethics Committee is only required for interventional studies. The study, including access to electronic follow-up in DNPR was approved by the Board at Aalborg University Hospital. As there is no contact with individual patients, approval from Ethics Committee is not mandatory.

## Results

### Study population characteristics

The study population comprises 314 referrals based on 307 patients referred to the NSSC-CPP in DC Farsø. Seven patients were readmitted to NSSC-CCP for various reasons, often because the patient was included in another CPP at first referral or did not show up. Ninety-five percent were referred by GPs with a median of 3.3 patients (range 0–10) per GP. Median age of patients was 65 years (range 18–92) and more women 165 (53%), than men.

### Diagnostic flow

#### Pathway trajectory

**Figure 1.**
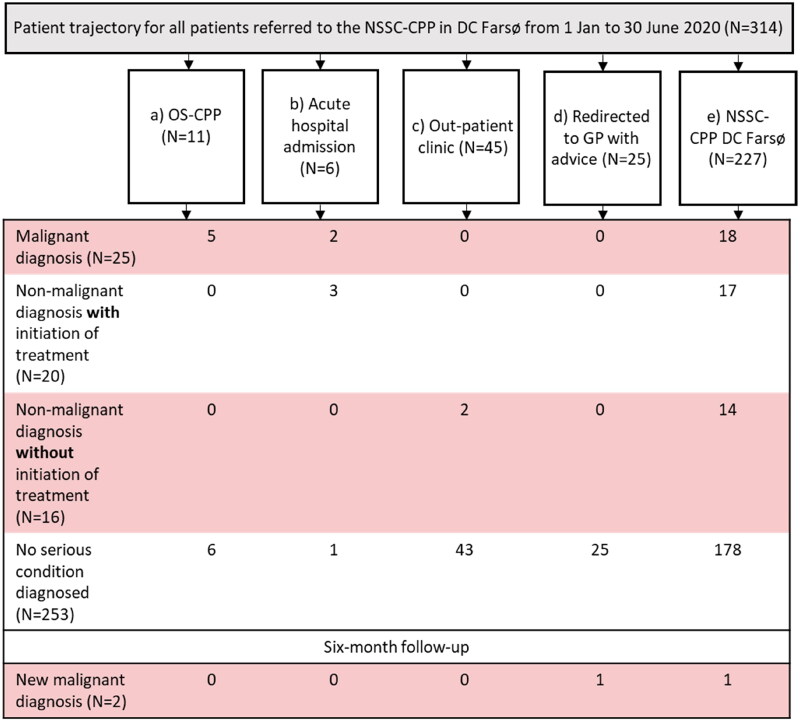
Patient trajectory and diagnostic outcomes for all referrals to The Cancer Patient Pathway for Non-specific Symptoms and Signs of Cancer (NSSC-CPP) in diagnostic centre, Farsø (DC Farsø) (*N* = 314).

**Figure 2.**
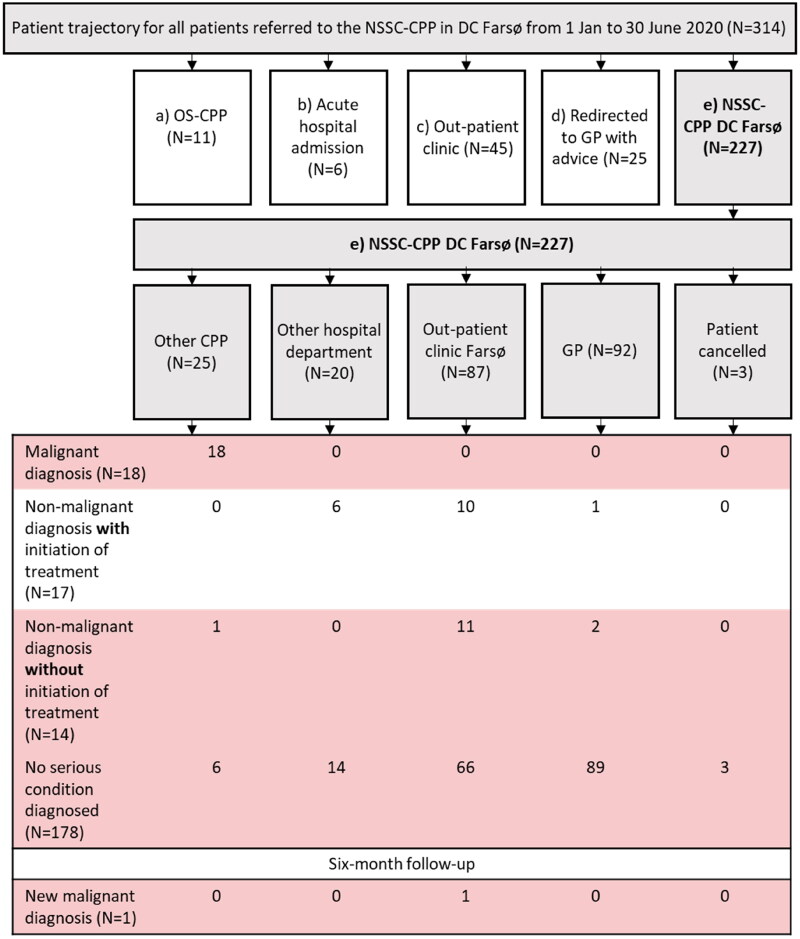
Detailed description of patient trajectory and diagnostic outcomes for patients with diagnostic work-up within The Cancer Patient Pathway for Non-specific Symptoms and Signs of Cancer(NSSC-CPP) in diagnostic centre, Farsø (DC Farsø) (*N* = 227).

As illustrated in Figure 1, 11 (4%) of the 314 referrals were redirected by the DC Farsø consultants to a relevant OS-CPP, 45 (14%) to out-patient clinics, 25 (8%) returned to GP with advice, six (2%) were referred to acute hospital admission while waiting for a consultation in DC Farsø, thus 227 (72%) for investigation in the NSSC-CPP in DC Farsø. After DC work-up in these 227 patients, 25 (11%) were redirected to relevant OS-CPP, 20 (9%) to other hospital departments, 87 (38%) to out-patient clinic Farsø, 92 (41%) returned to GP with advice and three patients (1%) cancelled their appointment, illustrated in Figure 2.

#### Symptoms and findings leading to referral

**Figure 3.**
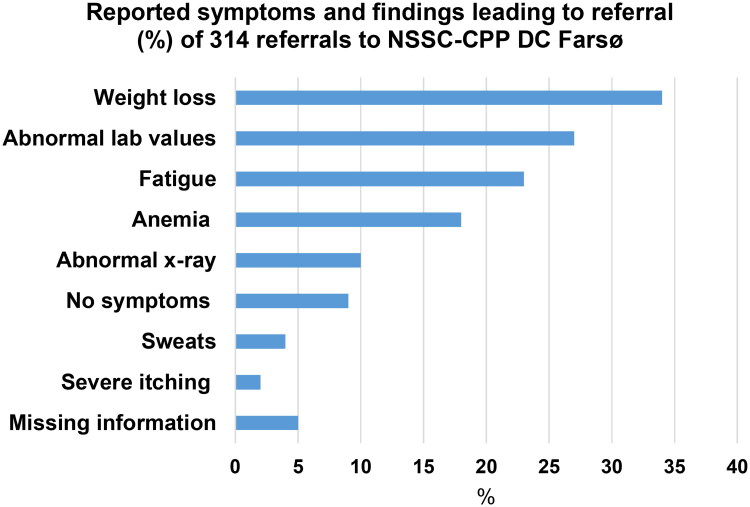
Reported symptoms and findings leading to referral (%) on 314 referrals to the Cancer Patient Pathway for Non-specific Symptoms and Signs of Cancer (NSSC-CPP) in diagnostic centre, Farsø (DC Farsø).

Symptom categories were predefined and referrals were often reported with more than one of the following symptoms thereby adding up to more than 100% (Figure 3): weight loss 106 (34%), fatigue 73 (23%), anaemia 55 (18%), abnormal laboratory values 86 (27%), abnormal x-ray 32 (10%), severe itching 5 (2%), sweats 14 (4%), no symptoms 28 (9%) and missing information on symptoms were reported for 15 (5%) referrals.

#### Diagnostic procedures

Of 314 referrals, CT scan was performed before DC Farsø in 117 (37%) referrals, CT scan in DC Farsø 98 (31%), X-ray 12 (4%), ultrasound 27 (9%), MRI 4 (1%), PET/CT 13 (4%), and no imaging was done for 87 (28%) referrals. Of the 314 referrals, gastroscopy was performed in 44 (14%) referrals, gastroscopy or colonoscopy just before DC Farsø referral 29 (9%), colonoscopy 7 (2%), sigmoidoscopy 1 (<0%), capsule endoscopy 1 (<0%) and in 249 (80%) referrals no endoscopies were performed. When the NSSC-CPP DC Farsø referrals (*n* = 227) were considered, the most used clinical procedure was CT scan performed in 196 (86%) of referrals before or within DC Farsø work-up.

#### Diagnoses after DC Farsø work-up and within a 6-month follow-up period

In total, 25 of 314 referrals (8%) were diagnosed with a new malignancy (Figure 1). Of the 314 referrals, 20 (6%) were diagnosed with a non-malignant but clinically relevant diagnosis with initiation of treatment, 16 (5%) a non-malignant diagnosis but no treatment initiated, and in 253 (81%) referrals no severe new condition was diagnosed, meaning that none of the above-described conditions was diagnosed. After completed diagnostic workups, two additional cancers were diagnosed within a six-month follow-up period (Appendix 1 for details). A detailed description of all diagnoses is presented in Table 1.

**Table 1.** Diagnoses for all 314 referred patients after work-up and within a 6-month follow-up period.

Malignancies (*n* = 2) in six patients referred to acute hospital admission		
	Lung	1
	Pancreas	1
Malignancies (*n* = 5) in 11 patients referred to an OS-CCP		
	Gastrointestinal tract	4
	Lung	1
Malignancies (*n* = 18) in 227 patients accepted for DC work-up		
	Lung	6
	Gastrointestinal tract	4
	Breast	2
	Renal	1
	Sarcoma	1
	Seminoma	1
	Lymphoma	2
	Polycythaemia vera	1
Non-malignant but clinically relevant diagnosis with initiation of treatment (*n* = 20)		
	Rheumatic polymyalgia and vasculitis	4
	Gastric ulcer	3
	Liver insufficiency and autoimmune hepatitis	2
	Pneumonia	2
	Myocardial infarction	1
	Atrial fibrillation	1
	Thyrotoxicosis	1
	Celiac disease	1
	Biliary diarrhoea	1
	Hemochromatosis	1
	Hepatitis C	1
	Pancreatic insufficiency	1
	Secondary polycythaemia	1
Non-malignant diagnosis without initiation of treatment (*n* = 16)		
	Infection with spontaneous remission	6
	Monoclonal gammopathy unknown significance	5
	Sarcoidosis	1
	Gallstone	1
	Menorrhagia	1
	Anaemia following gastric bypass	1
	Bleeding after biopsy in colon, spontaneous remission	1
New malignancies (*n* = 2) within 6-month follow-up		
	Cervical cancer with abdominal carcinosis	1
	Oesophageal cancer	1

## Discussion

### Main findings

Twenty-five (8%) of 314 referrals were diagnosed with cancer. In 253 (81%) referrals no severe new condition was diagnosed. We found that more than one-fourth of referrals to NSSC-CPP were redirected to other hospital departments or GPs. In patients accepted for work-up in DC Farsø, 17 (5%) were diagnosed with a non-malignant but clinically relevant diagnosis with initiation of treatment and 14 (4%) with a non-malignant diagnosis but no need for initiation of treatment. Two additional (1%) cancers were diagnosed within a six-month follow-up.

### Strengths and limitations

This study’s primary strength lies in its prospective design and detailed diagnostic flow tracking for all patients referred to the NSSC-CPP. Unlike many studies that overlook patients who drop out or are redirected, our comprehensive approach includes them, preventing selection bias common in such reports. A weakness is related to the small sample size, with 314 included referrals representing a half year of NSSC-CPP activity in DC Farsø. A greater sample size would provide a more valid description, for example, according to trends in types of cancer diagnosed. Additional, NSSC-CPP data from other DCs in Denmark would increase the extern validity of this study. Still, this study is valuable and unique due to its complete and long-term follow-up of all referrals to one Diagnostic Centre, ensuring high internal validity. Investigating NSSC-CPP referral effects on mortality would be interesting but requires a longer follow-up or larger sample size. Due to diverse NSSC-CPP organisations in Denmark, our findings may not directly apply elsewhere, lowering external validity. Nevertheless, our observations remain relevant for comparing practices within and outside Denmark.

### Comparison with existing literature

Other European studies on NSSC-CPP populations reported cancer diagnoses in 11%–22% [18–23], surpassing our 8%. Notably, most studies were conducted just after Denmark’s NSSC-CPP introduction (2012–2015). The NSSC-CPP facilitated easier GPs referrals for non-specific symptoms likely contributing to higher early malignancy detection. This phenomenon parallels initial rounds of screening programs, capturing both prevalent and incident cases, potentially explaining the elevated cancer rates observed in those studies.

The threshold for when to refer patients to diagnostic work-up might also be lowered due to current political and societal focus on cancer prevention and cancer diagnostics [24,25]. In comparison, a Swedish prospective study found that 22% of referrals to the Swedish NSSC-CPP were diagnosed with cancer, and in UK, this number was 8% [26,27]. We do not have an explanation for these geographical variations. It might be linked to variations in the referral criteria to the NSSC-CPP within each country or the fear of missing a malignant diagnosis. Increased focus in Denmark on the risk of receiving legal complaints it is likely that many doctors have changed diagnostic strategies in a more defensive direction.

Although data for this study was collected during COVID-19, we did not experience any remarkable changes regarding numbers of NSSC-CPP referrals or cancer incidence compared to previous years. In this study, CT scan was the most used diagnostic imaging as 68% received a CT scan or PET/CT. Jørgensen and colleagues found a similar number, which reported this percentages to be 69% [28]. Furthermore, when examining the NSSC-CPP DC Farsø referrals (*n* = 227) only, we found that 86% had a CT scan before or within the DC Farsø workup. CT scans are an essential tool within cancer diagnostics but might also increase incidental findings [29].

Only one study has investigated incidental findings in the context of the NSSC-CPP[30]. This study was based on data from the Oxfordshire’s, Suspected CANcer (SCAN) referral pathway, a UK version of the Danish NSSC-CPP [30]. Researchers found that 987 (95%) of patients referred to the SCAN pathway had at least one additional finding reported. Of the incidental findings, 140 (14%) were clinically significant. It means that people undergo extended testing, which for some is beneficial but for others might lead to overdiagnosis.

Future studies might investigate the effect of this high number of CT scans, including the radiation risk, incidental findings and overdiagnosis, to balance the benefits and harms of the NSSC-CPP.

### Implications for research, clinical practice and policy

No systematic review has been conducted; however, through a comprehensive review of the existing literature, we have discovered that no previous study in Europe has specifically examined the follow-up of patients who are referred to a diagnostic centre but do not proceed with the diagnostic work-up and are instead redirected to other healthcare facilities. However, this is an essential element if policymakers or others want to estimate the actual effect of the NSSC-CPP implementation. This study contributes to the existing literature by showing that more than one-fourth of patients first referred to the NSSC-CPP were redirected to other hospital departments or back to the GPs, which indicates that the inclusion criteria for the NSSC-CPP are not well defined or may not be agreed upon between health professionals. This study contributes with an important description of patient trajectories, which should be investigated in more detail in larger follow-up studies to assess benefits and potential harms of the NSSC-CPP. In this study, 25 patients were redirected to GPs without further diagnostic work-up. Of these, one patient was later diagnosed with new malignancy in the six-month follow-up. Although our sample size was small, it indicates that the consultants at the diagnostic centre most often make a qualified choice regarding which diagnostic trajectory is most suitable for the individual patient. At the same time, no serious condition was diagnosed in 253 (81%) referrals. However, it is a problematic and valuable judgement on how many ‘missed’ cancers can outweigh the number of patients that are spared for excessive testing and potential overdiagnosis. More prognostic studies assessing the consequences of potential false positives, incidental findings, overdiagnosis and overtreatment in long-term follow-up studies are needed to support such decisions. Also, future studies might assess whether NSSC-CPP diagnostic workup decreases overall mortality.

## Conclusion

This study is the first to follow the trajectory of all patients referred to the Cancer Patient Pathway for Non-specific Symptoms and Signs of Cancer (NSSC-CPP) in Denmark. Thereby, this study adds new knowledge on patients redirected to other hospital departments or GPs after NSSC-CPP referrals, which included more than one-fourth of referrals. Eight percent of referrals were diagnosed with cancer within the diagnostic process and two additional cancers were diagnosed within a 6-month follow-up period. This study contributes by providing an in-depth description of all patients regardless of later redirection, which is important in the future organisation of the NSSC-CPP.

## Appendix 1. Information on two patients in whom additional cancer was diagnosed within a 6-month follow-up period

Middle-aged male referred to NSSC-CPP fast track from the emergency room due to leucocytosis and intended 5 kg weight loss. However, based on two previous bone marrow aspirations, the haematologist had concluded that his bone marrow was normal with no need for further examination. And as the weight loss was intended, he was rejected from NSSC-CPP fast track. One month later, he was readmitted due to dyspepsia and additional weight loss. Endoscopy revealed oesophageal cancer.

Middle-aged previously healthy women presented with lower abdominal pain and 5 kg weight loss. She underwent colonoscopy, CT scan, gynaecological examination including ultrasound, MRI scan and extended blood panel, all of which turned out normal. Based on these normal findings, the DC work-up was stopped but she was followed regularly in the outpatient clinic. Three months later, she was admitted to the emergency ward for two days with intensified abdominal pain. CT scan showed ileus and later surgery revealed carcinomatosis. After several additional biopsies, the final diagnosis was cervical cancer.
